# Society Meetings

**Published:** 1903-04

**Authors:** 


### SOCIETY MEETINGS.

The National Association of U. S. Pension Examining Surgeons will hold its next annual meeting at Washington, D.C., under the presidency of Dr. William A. Howe, of Phelps, N. Y. The secretary, Dr. Wheelock Rider, of Rochester, is engaged in preparing a program of great interest and a large attendance is anticipated.

The American Urological Association will hold its next annual meeting at New Orleans, May 8 and 9, 1903. The president is Dr. Ramon Guitèras, of New York, and the secretary is Dr. F. C. Valentine, also of New York.

The American Association of Medical Editors will hold its next annual meeting at New Orleans, May 4, 1903, at which a large attendance is expected.

The Erie County Medical Association held its annual meeting March 9, 1903, in this city. The following-named were elected officers for the current year: president, Dr. Allen A. Jones; vicepresident, Dr. C. C. Frederick; secretary, Dr. Jacob Otto; treasurer, Dr. William I. Thornton.

---

The National Confederation of State Medical Examining and Licensing Boards will hold its next annual meeting at New Orleans, May 4, 1903. Dr. N. R. Coleman, of Columbus, O., is president, and Dr. A. Walter Suiter, of Herkimer, N. Y., is secretary.

---

The Buffalo Academy of Medicine held meetings during the month of March, 1903, as follows:

*Section on Surgery.*—Tuesday evening, March 3. Program: Provisional hemostasis in operations upon the head and neck, George Ryerson Fowler, of Brooklyn; discussion by Roswell Park and others.

*Section on Medicine.*—Tuesday evening, March 10. Program: Cardiac disease, H. C. Buswell; Local geographic conditions and population as bearing on the water supply and sewerage of Buffalo, A. L. Benedict.

*Section on Pathology.*—Tuesday evening, March 17. Program: On the cardiac and vascular complications of typhoid fever, W. S. Thayer, associate professor of medicine, Johns Hopkins University, Baltimore.

*Section on Obstetrics.*—Tuesday evening, March 24. Program: Why minor gynecological operations fail to give anticipated results, S. Goldberg; Some remarks upon ectopic gestation, C. C. Frederick.

